# A Few Additional Remarks on the Identity of the Electric and Nervous Fluids

**Published:** 1802-04

**Authors:** T. Purton

**Affiliations:** Member of the Royal College of Surgeons, in London


### *A few additional Remarks on the Identity of the Electric and Nervous Fluids, by* T. PURTON, *Member of the Royal College of Surgeons, in London.*

---

To the EDITORS of the MEDICAL and PHYSICAL JOURNAL.

GENTLEMEN,

AT a time when the minds of fo many Phyfiologifts are engaged in the inveftigation and difcoveries of the various phænomena of electricity, I think every man is bound to come forward with whatever might add to the fupport of, or will in the leaft tend to unravel, a fubject which, as yet, is involved in fo much obfcurity. How far the following additional remarks to thofe which I have before written,* may be confidered as a farther elucidation to thofe already advanced, I muft leave others to determine; but they may poffibly convey fuch an impreffion to other minds as may be productive of future difcoveries, and ultimately bring us to the truth.

My principal reafon for troubling you with any further remarks on this fubject, was occafioned by the perufal of a pamphlet

* Medical and Phyfical Journal, Vol. iv. p. 334, et feq.

phlet publifhed by the Rev. John Lyon, defcribing a very interefting account of the effects of lightning on the bodies of a man and four horfes, which were killed near Dover in Kent. The phænomena were truly interefting, for in three of the horfes the right ventricles of the heart were ruptured. In the other horfe the carotid artery on the right fide was ruptured, but the heart was not apparently injured. It is much to be regretted that the man was not opened.

Mr. Lyon, I think, proves to a demonftration, that the blood and not the nerves, was the conductor of the electric fluid, which certainly is not an inconfiderable addition in fupport of my general argument. Mr. Lyon does not appear to have underftood much of the circulation of the blood, or he would not have fuggefted the idea that the electric fluid was conveyed by the arteries to the right fide of the heart, which is not only directly contrary to the courfe and motion of the blood, but its termination in that cafe would have been into the left ventricle. I cannot attribute the immediate death of the horfes to the rupture of the ventricles, as I have not any doubt that this event would have been as inftantaneous if the whole vafcular fyftem had efcaped uninjured. The death of all animals from lightning, is, in my opinion, fully eftablifhed by facts and experiment, to be caufed by the concuffion and fudden fhock given to the brain; as to the accidental rupturing of the hearts in thefe inftances, it may be confidered as a fingular occurrence, owing probably more to the favourable pofition of the man and horfes to tranfmit the electric matter, than a general effect in all cafes.

If the arterial and venous circulation, which I have hinted at in my Paper on the phyfiology of the brain and nerves, are the collectors of the electric fluid, the phænomena attending the death of the horfes may be better explained; for the electric matter was paffing in the fame direction as it does in the ufual courfe to the common refervoir the brain. Time may determine how near I am to the truth; but I really think the courfe which the electric matter took, joining itfelf with that already accumulating in the body, ftrengthens very much my general doctrine. Is it at all improbable, that this penetrating matter, which by fome peculiar combination or modification is to produce the various phenomena in the nervous fyftem, may be accumulated through the medium of the circulation, being derived ab externo, by thofe innumerable pores with which the whole furface of the fkin abound, but principally by refpiration? I cannot help thinking that the oxygenation of the blood is in fome meafure connected with this very wonderful matter, either chemically or otherwife, in the circulation through the lungs; as as it is probable that the fenfation of the animal depends entirely upon the influence of refpiration; for we may find by daily obfervation that fenfibility feems to be impaired exactly in proportion as this function becomes defective, until, at length, they both are extinguifhed nearly at the fame inftant of time. How far the relation between oxygen and the electric fluid will bear an inveftigation is a matter worthy of the confideration of future phyfiologifts, and alfo the changes which evidently take place in the very properties of the blood, on the firft rufhing of the air into the cells of the lungs; probably the electric fluid owes its firft principal modification to this grand union with oxygenous matter. Thefe are certainly circumftances which require a very attentive refearch, as the refult of our inquiries may alfo point out, what conftitutes the diftinctive differences between fœtal and adult life. I think this cannot be denied, that one of the principal ufes of refpiration, is to keep up a certain energy or healthy organization in the brain and nerves, which qualify them fo eminently for the various faculties of perception and fenfation. This energy, probably, is nothing more or lefs than that the nerves and their appendages fhould conftantly poffefs a certain fpecies of electric influence, over and above what is accumulated in the brain for the different purpofes of the animal; fo that their remote and exterior ramifications fhould be kept in that continual ftate of fufceptibility which is effential to health.

The means which are ufually found effectual in refufcitating perfons apparently drowned or fmothered, infants ftill-born, &c. &c. appear to favour this theory; we firft of all produce action in the heart, but fenfation probably does not take place until the functions of the lungs are reftored. The ftate of the nerves before and after birth is certainly very different; they feem in utero to be merely the agents of motion, but after the blood has received the benefit of refpiration they immediately perform a much more important function.

The attrition and velocity of the circulation appear to me in every way fufficient for the purpofes of accumulating the electric fluid; which, probably, after it has undergone fome other changes in its circulation through the lungs, conftitutes that energy which we call nervous. The placenta acting as the fœtal lungs, is in every way calculated to produce fimilar changes in utero. The brain enclofed by its membranes and bony compages, might a priori be confidered as a proper refervoir for matter fo fubtle and diffufive in its nature. In the cafe of Dr. Monro, no doubt that the fpinal marrow enclofed by the theca vertebralis performed a fimilar office in utero; and fuppofing a child was born without a brain, fpinal marrow, and even a heart, of which there have been inftances, the arteries and nerves under the above theory would have anfwered every purpofe. Therefore, under this idea, the nerves have nothing to do with the office of accumulating the electric matter, but of tranfmitting the fame, after a certain modification, to all parts of the body, for the purpofes of fenfation and mufcular motion.

The courfe of the circulation being furrounded by the beft non-conductors, may be confidered as acting fimilar to the glafs cylinder in an electric machine, and the brain as the Leyden phials; and how could nature fuggeft a more admirable contrivance either to confine or prevent a difperfion of this fluid, or to conduct it more rapidly, than is found in the human body? Where is there a better non-conductor than the fat? and, on the other hand, where can there be found better conductors than the nerves and blood?

If it fhould be thought that I treat a fubject of fo much importance too lightly, by comparing the nervous fyftem to an electric machine, I can only fay, that it is far from my intention to do it; but in a matter of fo much obfcurity, how can we poffibly arrive at the truth, unlefs all our reafoning and experiments are grounded upon an analogy with well eftablifhed facts.

If the prefent Inquiry fhould terminate in giving place to a theory ftill more defenfible, it muft furely be to fomething which will bear a very great analogy to the matter of electricity; for in how many points do thefe two very fimilar fluids agree? yet there are ftill differences which, I muft acknowledge, are at prefent not explicable; but I entertain the moft flattering hope that the time will foon arrive when this fubject will be far better underftood. After a very principal part of the circulation of the blood was found out, a confiderable fpace of time elapfed before the whole was completely difcovered; and when we confider what a little was left to be done, our furprife muft be great indeed, to find, that nearly a century paffed before the difcovery was perfectly eftablifhed.

There are many natural productions, which bear fo clofe a refemblance to each other in their very component parts, that we cannot help proclaiming them modifications of one and the fame principle. May not the electric matter be fo modified and changed by its circulation or otherwife, through the various parts of the body, as to be totally altered in many of its conftituent properties?

My mind is impreffed with fuch conviction, that I cannot forbear thinking, we are in the right path to truth; therefore, let me exprefs a wifh, that Phyfiologifts will ftill turn their attention, attention, very earneſtly, to this moſt important ſubject; and if any thing which I have ſuggeſted ſhould be directly or indirectly the means of making it more clear, I ſhall conſider my time well employed.

I am, &c.

*Alcester, March* 1, 1802. T. PURTON.

